# Non-Suicidal Self-Injury and Its Relationship with Family Psychological Function and Perceived Social Support among Iranian High School Students

**DOI:** 10.34172/jrhs.2020.04

**Published:** 2020-02-27

**Authors:** Hossein Nemati, Mohammad Hasan Sahebihagh, Mahbobeh Mahmoodi, Akbar Ghiasi, Hossein Ebrahimi, Shirin Barzanjeh Atri, Asghar Mohammadpoorasl

**Affiliations:** ^1^Department of Community Health Nursing, School of Nursing & Midwifery, Tabriz University of Medical Sciences, Tabriz, Iran; ^2^HEB School of Business and Administration, University of the Incarnate Word, San Antonio, TX, USA; ^3^Department of Psychiatric Nursing, School of Nursing & Midwifery, Tabriz University of Medical Sciences, Tabriz, Iran; ^4^Research Center of Psychiatry and Behavioral Sciences, Tabriz University of Medical Sciences, Tabriz, Iran

**Keywords:** Non-suicidal self-injury, Family, Social support, Adolescent

## Abstract

**Background:** Non-suicidal self-injury (NSSI) has become one of the serious public health concerns among adolescents. Factors like family and social environment of adolescents may be important determinants of the NSSI. This study aimed to investigate the relationship between family psychological function and perceived social support with the NSSI experience among adolescents.

**Study design:** A cross-sectional study.

**Methods:** Overall, 4216 high school students (15-18 yr old) of Tabriz City, northwestern Iran were selected using multi-stage cluster random sampling method in October and November 2017. Participants completed survey including demographic characteristics, NSSI status, Iranian family psychological function, and perceived social support. After six months, NSSI status was reassessed. The data were analyzed using logistic regression model.

**Results:** 8.5% of the students had NSSI experience. In addition, the weak family psychological function increased the odds of experiencing the NSSI by 13 times compared to the strong psychological function (OR = 13.15, 95% CI: 7.19, 23.80). Besides, the low level of perceived social support increased the odds of experiencing the NSSI by about 7 times compared to the high perception of social support (OR= 6.67, 95% CI: 4.01, 11.11).

**Conclusion:** Low levels of psychological functioning of the families and perception of social support significantly can increase the odds of experiencing the NSSI among adolescents. Therefore, special attention should be paid to these factors in the development of relevant preventive programs in adolescence period.

## Introduction

Non-suicidal self-injury (NSSI) is a complex behavior, in which a person induces damage to one of his/her body tissues directly and deliberately without suicidal intent^1^. This behavior is considerably more prevalent during adolescence period and has become one of the most important concerns of public and health policymakers. NSSI during adolescence is one of the determinants of psychological disorders and suicidal attempts in later years of life^2^. A comprehensive systematic review reported the prevalence of NSSI in adolescence between 7.5% and 46.5%^3^. In another review study, the prevalence of NSSI in Iranian adolescents was 4.3% to 26.8%^4^.

Numerous factors were associated with incidence of NSSI behaviors among adolescents such as gender, emotional and psychological disorders, drug abuse, and experiences of violent behaviors during childhood, stressful family environment, and poor parent-child relationships ^5^. Family is one of the first environments that provides physical, psychological, and social health in addition to financial and economic support. A family has psychological functions that provide a safe and secure environment to meet the psychological needs of its members^6^. Family conflicts as well as poor family bond are associated with numerous destructive behaviors of adolescents such as NSSI^7^.

Adolescents with sufficient social supports from family, friends, and other key people in their life have better mental health, and less high-risk behaviors^8^. Social support is investigated both as received and perceived social support. The perceived social support is mental expectations and evaluations of a person from the available, appropriate, and sufficient social network in required cases. Furthermore, it indicates the perceptions of an individual who is loved, cared, respected, and considered as a part of a social network. The perception of social support is particularly important and may have psychological benefits for people who are dealing with physical, psychological, and social stressful events ^9^.

The social motivations as well as family and social environments may be particularly important at the initiation and repetition of NSSI ^10^. There are many hypotheses about the impact of family and social factors on NSSI, but there is limited information about the impact of psychological functions of family and perceived social support with the experience of NSSI among adolescents.

Given the existence of instrument for measuring psychological function of the Iranian family driven from Iranian culture and properly covers the components of the Iranian family, we aimed to investigate the relationship between psychological functions of family and perceived social support with experience of NSSI among adolescents of Tabriz City (Northwest of Iran).

## Methods

This study was conducted in Tabriz, northwestern Iran in October and November 2017. According to official reports, 50790 students (25339 males and 25451 females) in 334 schools (163 for males and 171 for females) in 5 districts were studying in the high schools in Tabriz. The multi-stage proportional cluster sampling method was used. First, the number of students in each district was calculated, and the number of samples from each district was specified as proportion of total number of students in that district. Second, 60 schools were selected using simple random sampling from the high schools and vocational schools to achieve the specified quota. One or multiple classes were selected from each of selected classes. Overall, 156 classes (75 for males and 81 for females) were included in study by considering students’ major and entered to the study. Finally, 4216 high school students were selected. The exclusion criteria in this study were orphaned adolescents or adolescents under the custody of relatives, failure to complete the questionnaire, and experience of crisis over the past year (such as disrupted families).

Required permits were obtained from Tabriz University of Medical Sciences (Ethics Code: IR.TBZMED.REC.1396.274) and Organization of Education of East Azerbaijan Province.

Participants completed a self-administered multiple-choice anonym questionnaire between Nov 2018 and Dec of 2018. Six months later, the same questionnaire (after excluding the unnecessary parts) was distributed to the same students in order to study incidence of the NSSI. The questionnaire contained demographic characteristics, the NSSI status, Iranian family psychological function, and perceived social support.

### The NSSI measurement

NSSI was measured using open-ended questions. Students were asked if they had ever experienced self-injury (intentional damage to the body). If the answer was yes, they were asked to specify the type of injury. During review of the completed surveys, some behaviors like eating disorders, masturbation, and tattooing were removed because they were not considered self-injury.

### Iranian Family psychological function

The Iranian family psychological function questionnaire contains 89 questions to evaluate psychological functions of families. This questionnaire includes 12 dimensions: communication, progress, emotional literacy (love and affection), spirituality and religion, leisure, alliance, structure and organization, sense of security, conflict resolution, and devotion to relatives, independence, and control. The minimum possible score is 89 and the maximum score is 623. The score 89-237 indicates weak function, 237-356 shows moderate function, and score above 356 indicates strong psychological function of the family. The validity and reliability of this questionnaire were confirmed in Iran ^6^.

### Perceived social support questionnaire

The perceived social support questionnaire was designed, which contains 12 items and evaluates three dimension of supports including support from family, friends, and other important people around students ^11^. Each dimension has four questions. The respondents indicated their opinion on each question on a 5-point scale from 1 for totally disagree to 5 for totally agree. The questions were Likert scaled questions with five choices (1 totally disagree, 2 disagree, 3 neutral, 4 agree and 5 totally agree). The score range was from 12 to 60. The score 12 to 24 shows low perception, 24 to 36 moderate perception, and higher than 36 corresponds for high perception of social support. Cronbach's alpha coefficient of 0.89, 0.86, and 0.82 showed for three dimensions of family, friends, and other important people in life respectively ^12^.

### Socioeconomic status

The socioeconomic status of students was estimated based on the father's education, mother's education, household assets(including durable goods such as refrigerators, freezers, washing machine, dishwasher, microwave, vacuum cleaner, computer, car, etc.), living place, and household income by using principal component analysis (PCA). Since the data in PCA are quantitative, in this study we used polychoric PCA. According to the score obtained from PCA, the students were classified in five equal levels of socioeconomic status as very high, high, moderate, low, and very low.

Data were analyzed using SPSS-24 software (Chicago, IL, USA). Descriptive statistics like frequency and percentage for categorical variables and mean and standard deviation for continuous variables were reported. To determine the relationship between dependent and independent variables, t-test, Chi-square, and logistic regression models were used.

## Results

Out of 4216 students, 3966 participants (94.1%) completed the questionnaire. Out of non-respondents, 221 (5.2%) did not completely were absent at survey completion day, and 29 (0.7%) were reluctant to complete the questionnaire. In terms of gender distribution among respondents, 1868 (47.1%) were male and 2098 (52.9%) were female. The mean age of the respondents was 15.95 ±0.74 (age range 15-18). In addition, 1350 (34.0%) of respondents were in sciences field, 1009 (25.5%) were in the technical and vocational field, 817 (20.6%) were social and human sciences, and 790 (19.9%) were mathematics.

The prevalence of NSSI at the beginning of the study was 6.2% (95% CI: 5.48, 6.99) and its incidence over the period of 6 months was 3.4% (95% CI: 2.72, 3.82). Out of 3966 students, 336 (8.5%, 95% CI: 7.65, 9.38) had NSSI experience.

The NSSI status at various levels of demographic variables, family psychological function, and perceived social support is shown in Table 1. Accordingly, all the variables had significant relationship with the NSSI. Moreover, 60.7% of individuals with weak family psychological function experiences NSSI, while 27.4% and 4.3% of individuals with a moderate and strong psychological function exposed experienced this NSSI respectively. Furthermore, 46.9% of participants with low perceived social support experienced NSSI. On the other hand, only 14% and 4.3% of individuals with moderate and high perception of social support experienced the NSSI respectively.

**Table 1:** Non-suicidal self-injury status at different levels of demographic variables, family psychological function, and perceived social support

**Categorical variable**s	**Non-suicidal self-injury**				**Number**	**Total** **Percent**	***P*** **value**
	**Yes**		**No**				
	**Number**	**Percent**	**Number**	**Percent**			
Gender							0.013
Male	180	9.6	1688	90.4	1868	47.1	
Female	156	7.4	1942	92.6	2098	52.9	
Total	336	8.5	3630	91.5	3966	100	
Age (yr)							0.001
15	62	5.4	1084	94.6	1146	29.0	
16	151	8.0	1735	92.0	1886	47.7	
17	117	13.5	752	86.5	869	22.0	
18	6	11.8	45	88.2	51	1.3	
Socio-economic status (SES)							0.005
Very low	72	9.7	670	90.3	742	20.2	
Low	66	8.8	684	91.2	750	20.4	
Middle	50	6.8	688	93.2	738	20.0	
High	47	6.5	680	93.5	727	19.8	
Very high	81	11.2	641	88.8	722	19.6	
Field of study							0.018
Mathematics	76	9.6	714	90.4	790	19.9	
Empirical sciences	123	9.1	1227	90.9	1350	34.0	
Humanities	47	5.8	770	94.2	817	20.6	
Technical and vocational	90	8.9	919	91.1	1009	25.5	
Living with parents							0.047
Both	310	8.2	3465	91.8	3775	95.4	
Single parent	22	12.2	159	87.8	181	4.6	
Iranian Family psychological function							0.001
Weak	71	60.7	46	39.3	117	3.5	
Moderate	84	27.4	223	72.6	307	9.1	
Strong	126	4.3	2836	95.7	2962	87.4	
Multidimensional perceived social support							0.001
Low	99	46.9	112	53.1	211	5.7	
Moderate	90	14.0	551	86.0	641	17.2	
High	125	4.3	2752	95.7	2877	77.1	
**Continuous variables**	**Mean**	**SD**	**Mean**	**SD**	**Mean**	**SD**	***P*** **value**
Previous year average grades	17.76	1.85	16.70	2.54	17.67	1.94	0.001

To test the relationship between family psychological function and perceived social support with the NSSI, the univariate and multivariate logistic regression analysis was used (Table 2). In this study, we controlled for gender, age, socioeconomic status, field of study, living with parents, and previous year average grades. The lower levels of family psychological function and perceived social support in the univariate model were significantly related to NSSI experience. By controlling for confounders, the moderate family psychological function increases the odds of experiencing the NSSI 2.5 times compared to the strong family psychological function (OR= 2.51, 95% CI: 1.45, 4.34) and the weak family psychological function increases the odds of experiencing the NSSI 13 times compared to the strong function (OR= 13.15, 95% CI: 7.19, 23.80). The moderate level of perceived social support increases the odds of experiencing the NSSI 2 times compared to the high perception of social support (OR= 2.08, 95%CI: 1.30, 3.33) and the low perceived social support increases the odds of experiencing the NSSI 7 times compared to high perception (OR = 6.67; 95% CI: 4.01, 11.11).

**Table 2:** Logistic regression between family psychological function and perceived social support

**Variables**	**Univariable analysis**		**Multivariable analysis**	
	**OR** ^(^ **95% CI)**	***P*** **value**	**OR (95% CI)** ^a^	***P*** **value**
Psychological functioning of the family				
Strong	1.00		1.00	
Moderate	4.09 (2.61, 6.41)	0.001	2.51 (1.45, 4.34)	0.001
Weak	34.48 (23.25, 52.63)	0.001	13.15 (7.19, 23.80)	0.001
Perceived social support				
High	1.00		1.00	
Moderate	5.40 (3.81, 7.69)	0.001	2.08 (1.30, 3.33)	0.002
Low	19.60 (14.08, 27.02)	0.001	6.67 (4.01, 11.11)	0.001

^a^ Adjusted for gender,age and socioeconomic status, field of study, living with parents, and previous year average grades

## Discussion

This study aimed to determine the relationship between family psychosocial function and perceived social support with experience of NSSI among students age 15 to 18. The prevalence of NSSI at the beginning of the study was 6.2% and its incidence over six months was 3.4%; about 8.5% of the students experienced NSSI. Among states of the US, the prevalence of NSSI was reported 6.4% to 14.8% among high school males and 17.7%-30.8% among high school females ^13^. Another study in 11 European countries reported the prevalence of NSSI 27.6% ^14^. In China, the prevalence of NSSI among high school students was 22.4% ^15^. Therefore, the prevalence of NSSI in our study was less than other similar studies. This difference may be due to NSSI definition and the assessment tool in different studies.

Family is the main social group for many people. Inside family, individuals feel safe and comfortable. In fact, one of the basic functions of family is psychological function, which improves mental health status of its members and reduces the probability of exposure to high-risk behaviors like NSSI. Among various factors affecting mental health/disorders, psychological function of family and emotional aspects of relationships among family members play a central role ^6^. Family dysfunction was associated with NSSI among adolescents ^16^. The poor family function is one of the determinants ofNSSI incidence during childhood and adolescence ^17^. The findings of current study showed a significant relationship between family psychosocial function and NSSI experience. Weak and moderate psychosocial functions of family compared to strong function increases the odds of NSSI experience among adolescents. Poor family interactions and lack of positive emotional relationship among members of a family plays an important role in the incidence of NSSI ^18^. In addition, adolescents with self-injury behaviors had emotional problems mainly caused by family conflicts ^19^. Likewise, the lack of warm parent-child relationships and intra-family conflicts increased the likelihood of NSSI exposure among adolescence. Moreover, it is essential to focus on improving relationship among family members in any type of family therapy intervention ^20^. Attention to family psychological functions, improving interactions among family members, and families’ empowerment in this field, can promote the effectiveness of NSSI prevention programs during adolescence.

The low levels of perceived social support increased the odds of NSSI experience among adolescents. Individuals with self-injury behaviors perceived less social support used numerous methods to damage their body ^21^. There was a significant relationship between perceived social support and incidence of NSSI ^22^. Family and friends were among the main sources of social support ^8^. People with the NSSI behavior reported poor perception of social support from family members. Moreover, the lack of social support from family increases vulnerability to incidence of the NSSI behaviors ^10^. The effect of social support from friends and peers could be different among adolescents; for example, the social support of peers might reduce the incidence of NSSI for some people, on the other hand, it might increase the incidence of NSSI for others ^23^. 38.3% of adolescents initially learned about NSSI from their peers ^24^. Adolescents with low perception of social support might engage in NSSI behaviors as an instrument to acquire support that is more emotional or attention from friends and family.

The results of this study showed that among individuals who were part of two-parent family were less likely to engage in NSSI behaviors compared to those in single parents. Children who are living in two-parent families have better quality of life, had more access to health care, and exposure to less emotional and behavioral problems than children who are living in single-parent families ^25,26^. In two-parent family, both parents- father, and mother play an important role in managing behavioral problems and improving the health status of their children.

Like many empirical studies, limitations were associated with current study. One of the main limitations of this study was measuring self-injury experience using the self-report method. In this method, participants may exaggerate symptoms in order to make their situation seem worse, or they may under-report the severity or frequency of symptoms in order to minimize their problems. Moreover, they might simply be mistaken or misremember the material covered by the survey. In addition, the family psychological function and perceived social support were measured at the beginning of the study and their potential variation was not measured over time.

## Conclusion

Lower levels of family psychological function and perceived social support increased the odds of NSSI among adolescents. Family-centered interventions with the aim of empowering families in terms of psychological functions as well as promoting of perceived social support may improve the effectiveness of NSSI prevention programs in the adolescent period.

## Acknowledgements

We would like to thank all participants in this research. We could like to thank Vice-chancellor for Research and Technology of Tabriz University of Medical Sciences for financial support and Organization of Education of East Azerbaijan Province for cooperation in this study.

## Conflict of interest

None declared.

## Funding

This study was supported by Tabriz University of Medical Sciences.

## Highlights

- Low levels of family psychological function increase odds of experiencing the NSSI
- Adolescents with low perception of social support might engage in NSSI behaviors.
- Adolescents who are living in two-parent families were less engage in NSSI.
